# Public health research in the UK: in response to McCarthy *et al*.

**DOI:** 10.1093/pubmed/fdu088

**Published:** 2014-11-13

**Authors:** Andrew Cook, Hannah Dorling, Liz Ollerhead, Matthew Westmore, Phil Taverner

**Affiliations:** 1 Wessex Institute, University of Southampton, Southampton, UK; 2 University Hospital Southampton NHS Foundation Trust, Southampton, UK; 3 National Institute for Health Research Evaluation, Trials and Studies Coordinating Centre (NETSCC), Southampton, Hampshire, UK

Sirs,

We read with interest ‘Public health research in the UK: a report with a European perspective’ by McCarthy *et al.* published in June 2014. We are conscious that, since the study was undertaken in 2010, the public health research landscape in the UK has developed considerably. A number of programmes were set up in 2009 which now have strong portfolios of public health research. All parts of the National Institute for Health Research (NIHR) support research in public health to provide significant opportunities for researchers and evidence for practitioners and decision-makers (www.nihr.ac.uk/publichealth). Public health research is particularly funded by the NIHR through the Public Health Research programme, focusing on interventions outside of health care, and the School for Public Health Research. The Health Technology Assessment programme evaluates NHS interventions, while Programme Grants for Applied Research supports NHS research delivering findings with early practical application. While the remit varies by programme, covering England or the UK, the application of research findings could extend across Europe. Research for public health is also supported by other Department of Health funding schemes, such as the Policy Research Programme and other funding bodies; for example, a recent option for early-phase intervention research is the Medical Research Council's Public Health Intervention Development scheme. NIHR outputs are captured in a suite of five peer-reviewed, open access journals for anyone to use (http://www.journalslibrary.nihr.ac.uk/). We welcome contributions to identify research needs, deliver research to answer key questions and bring new evidence back into practice to strengthen public health research in the UK for the future.

